# Zoospore diversity and sexual reproduction in the lichen‐forming genus *Trebouxia*: From neglected evidence to new facts

**DOI:** 10.1111/plb.70042

**Published:** 2025-06-03

**Authors:** E. Boccato, D. Porrelli, C. G. Ametrano, F. Candotto Carniel, M. Tretiach

**Affiliations:** ^1^ Department of Life Sciences University of Trieste Trieste Italy

**Keywords:** Chlorophyta, flagellate cell, gamete, SEM, sexuality, *Trebouxiophyceae*

## Abstract

- *Trebouxia* is one of the most frequent genera of green microalgae that occur as photobionts in lichens. However, its life cycle is still poorly understood. The contradictory information about the flagellate cells impairs our knowledge of possible sexual reproduction. The aim of this study was to investigate the behaviour and fate of flagellate cells in four *Trebouxia* species, to better understand their role in the life cycle.
- Axenic cultures of *Trebouxia* were grown under controlled conditions. The cell cycle of flagellate cells was observed using a novel technique for real‐time monitoring with light microscopy, validated in more detail by scanning electron microscopy (SEM). Additionally, a molecular approach was used to investigate genomic evidence of sexual reproduction.
- The zoospores had two significantly different morphotypes, elongated and subspherical, suggesting that this dimorphism should be recognised in future species descriptions. Fusions of elongated gametes were observed in three species, with formation and development of a zygote documented in one case. SEM images provided further evidence of plasmogamic events in unprecedented detail. Molecular analyses confirmed the presence of key meiotic genes in eight genomes and one transcriptome of *Trebouxia*, providing further strong evidence of sexual reproduction in this genus.
- This study provides a new method to monitor the fate of flagellate cells over time which allowed demonstration of the presence of two types of flagellate cell: zoospores with two well‐defined morphologies which are involved in asexual reproduction, and gametes involved in sexual reproduction.

## Graphical abstract

This study clarifies the role of flagellate cells in four *Trebouxia* species. Using innovative techniques, we could distinguish between zoospores and gametes, providing the first detailed evidence of sex.

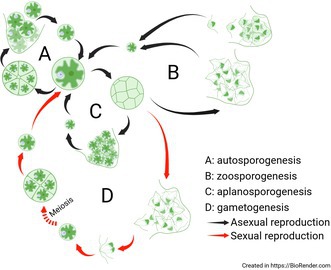

## INTRODUCTION

*Trebouxia* Puymaly (Trebouxiophyceae, Chlorophyta) is a genus of unicellular, aero terrestrial green microalgae, whose representatives are the photosynthetic partner (i.e. photobiont) in ca. 50% of all lichen species (Muggia *et al*. 2017). As a photobiont, *Trebouxia* provides essential photosynthetic products to the fungal partner (i.e. mycobiont) (Honegger 1997). Together, these two partners can colonise a wide range of habitats, from deserts to alpine and polar environments (Kranner *et al*. 2008), from which they would otherwise be precluded. Although the existence of Trebouxias as free‐living organisms was controversial (e.g. Ahmadjian 2001), modern molecular approaches have confirmed their presence as dominant free‐living species in some environments (Veselá *et al*. 2024). This genus has been one of the most studied photobionts since its first isolation from lichen thalli (Famintzin & Baranietzky 1867). From that time onwards, studies on this genus have used material isolated from lichens then cultivated as aposymbionts (e.g. Warén 1920; Jaag 1929; Starr 1955). Studying *Trebouxia* in culture is crucial, as the key species‐identifying traits, e.g. chloroplast morphology or specific life cycle stages, can be difficult—if present—in the lichenised form (Friedl & Büdel 2008). Species of *Trebouxia* can reproduce asexually via autospores, zoospores and aplanospores (terminology according to Tschermak‐Woess 1989), which have or lack a cell wall. Autospores are relatively large daughter cells derived from division of mother cells (i.e. autosporangia, which typically contain 4, 8 or 16 autospores) and have a cell wall. Zoospores are flagellate, originate from large mother cells (i.e. zoosporangia, which typically contain 32, 64 or 128 zoospores) and have no cell wall; these can actively swim in aqueous media. Aplanospores are relatively small daughter cells that arise from the arrested release of zoospores when still present in the mother cells (i.e. aplanosporangia, which typically contain 32, 64 or 128 aplanospores), and have a cell wall. As mentioned above, non‐motile stages of the *Trebouxia* life cycle have been extensively studied as they offer important taxonomic traits. Moreover, modern integrative taxonomic approaches (molecular data combined with chloroplast morphology and pyrenoid ultrastructure) favour the study of mature vegetative cells (e.g. Muggia *et al*. 2020; Bordenave *et al*. 2022; De Carolis *et al*. 2022), as the pyrenoid is poorly developed at other life stages and “only vegetative cells which are not preparing for protoplast division can be used for comparisons” (Friedl 1989), making the motile stage of the life cycle less taxonomically relevant.

Interestingly, although development stages of flagellate cells of *Trebouxia* in a lichen thallus have seldom been documented (Slocum *et al*. 1980), motile zoospores released from lichen thalli have never been reported, leading to the assumption that production of flagellate cells is somehow suppressed in the symbiosis due to the integrated nature of the relationship (Law & Lewis 1983). The presence of zoospores in culture, however, has long been documented (Woronine 1872) and frequently mentioned in species descriptions. Their ultrastructure is thoroughly studied (Slocum *et al*. 1980; Peveling & König 1985; Melkonian & Peveling 1987; Barreno *et al*. 2022) but, unfortunately, study of the motile stage of *Trebouxia* has been continually neglected over the years. This has affected the information available on sexual reproduction in *Trebouxia*. While sexual reproduction is common and confirmed in many members of the Chlorophyceae and Ulvophyceae (Veselá *et al*. 2024), it has generally been considered rare or absent in Trebouxiophyceae (Fučíková *et al*. 2015). In green algae, sexual reproduction occurs by isogamy, anisogamy or oogamy, always involving a flagellate life stage (Melkonian 1980; Nozaki 1983; Sekimoto 2017). However, evidence of sexual reproduction in Trebouxiophyceae has been observed in very few genera (Friedl & Rybalka 2012; Škaloud *et al*. 2015), and some authors even doubted that sexual reproduction is present in *Trebouxia s. str*. (Gärtner 1985; Friedl & Büdel 2008). Recent genomic and transcriptomic analyses (Blanc *et al*. 2010; Fučíková *et al*. 2015) have shown the presence of meiotic genes in several representatives of Trebouxiophyceae, suggesting that they possess the genetic apparatus for meiosis, and thus for sexual reproduction (Schurko & Logsdon 2008). In *Trebouxia*, some researchers have provided illustrations of fusion of putative gametes indistinguishable from zoospores (Warén 1920; Jaag 1929; Ahmadjian 1960, 1967; Komárek & Fott 1983), although some molecular evidence of recombination in *Trebouxia* populations was provided by Kroken & Taylor (2000). A further step forward was taken in the very recent publication of Gazquez *et al*. (2024), who investigated the life cycle of a single species, *T. lynniae*, focusing on the flagellate cells. Using cytofluorimetry and confocal techniques, they found that all mature vegetative cells had twice the relative DNA content of the flagellate cells (2c and 1c DNA content, respectively), with the exception of some flagellate cells, which had a 2c DNA content and were interpreted as zygotes. The authors concluded that *T. lynniae* has a diploid genome, sexual reproduction and a diplontic life cycle. Accordingly, they claimed that, in this species, all flagellate cells are gametes (otherwise mature 1c cells would have been detected), thus challenging current understanding of the vegetative development of flagellate cells as demonstrated in many other congeneric species (e.g. Peveling & König 1985; Melkonian & Peveling 1987; Tschermak‐Woess 1989; Friedl 1993). Although these results certainly add to knowledge of sexual reproduction in *Trebouxia*, they are difficult to reconcile with established notions on asexual reproduction. Therefore, the nature of flagellate cells in the life cycle of *Trebouxia* is still unclear and further studies are clearly needed. A major limitation of previous studies was the methods used for observation: the common practice of fixing flagellate cells for light microscopy (LM) or confocal microscopy restricts the observed cells to a static state and prevents real‐time observation of their behaviour or development. In addition, study of the flagellate stages of *Trebouxia* traditionally relies on LM and transmission electron microscopy (TEM), whereas scanning electron microscopy (SEM) is widely used in other green algae to obtain detailed information on the motile stages (e.g. Bråten 1971; Mogi *et al*. 2008; Innami *et al*. 2022). To fill this gap, we examined the flagellate cells of four *Trebouxia* species belonging to three of the five main clades recognised in this genus. We were not only able to characterise the motile stages of these *Trebouxia* species, but also to follow their real‐time behaviour and development by introducing a novel technique for real‐time monitoring under LM and a new protocol for acquisition of SEM images. With the present study, we aim to gain new insights into the flagellate cells of *Trebouxia* and thus improve our understanding of its life cycle, which has been poorly understood to date.

## MATERIAL AND METHODS

### *Trebouxia* strains and culture conditions

Axenic cultures of *Trebouxia decolorans*, *T. gelatinosa* and *T. vagua* were obtained from the Culture Collection of the University of Trieste (Italy), isolated from the lichens *Xanthoria parietina* (L.) Th. Fr., *Flavoparmelia caperata* (L.) Hale and *Bagliettoa marmorea* (Scop.) Gueidan & Cl. Roux. Axenic stock cultures of *T. angustilobata* (SAG 2204) were obtained from the Culture Collection of Algae of the University of Göttingen (Germany). All cultures were grown on solid *Trebouxia* medium (TM; 1.5% agar) (Ahmadjian 1973) in Microbox Junior 40 vessels (Combiness, Sac O_2_, Nevele, BE) and subcultured every 3 weeks. Vessels were kept in a thermostatic chamber at 16 ± 1°C and 23 ± 1 μmol photons m^−2^ s^−1^ with a light/dark regime of 12 h/12 h.

### Morphological analysis of flagellate cells using light microscopy

After 3 weeks under the above‐mentioned culture conditions, ca. 0.4 g of cells of each species were transferred from solid TM to 15 mL of liquid Bold's Basal Medium (BBM) (Nichols & Bold 1965). To obtain a homogeneous suspension, the cells were extruded from a needleless syringe through a nylon net of 40 μm mesh. An aliquot of 5 μL algal suspension was inoculated over a thin layer of solid BBM (1.5% agar) spread on a microscope slide (Menzel Gläser; Thermo Fisher Scientific, Waltham, USA). Slides were not covered with glass coverslips to prevent the spread of flagellate cells but were marked to find the same observation points through the coordinate system on the microscope stage over the following days. Slides were then transferred to Petri dishes lined with moist adsorbent paper to maintain humidity then placed in a thermostatic chamber at 20 ± 1°C and 23 ± 1 μmol photons m^−2^ s^−1^ with a light/dark regime of 12 h /12 h. Light was provided by white LED strips (3000 K; Life Electronics, Riposto, IT). The presence of flagellate cells was checked daily for a maximum of 14 days using a Primostar 3 optical microscope (Zeiss, Oberkochen, DE) and, when present, images and videos were recorded using a microscope camera (Axiocam 208 colour; Zeiss). A morphometric characterisation of flagellate cells was conducted using frames from the videos where flagellate cells were still moving, in focus and with the axes perpendicular to the optical axis of the microscope. To characterise the shape of each flagellate cell (*n* > 150 per species), the area, perimeter, primary and secondary axis of the best fitting ellipse (i.e. major and minor axes, respectively; *Fit Ellipse* option selected), circularity, defined as $4\pi \times \frac{\text{Area}}{{\text{Perimeter}}^{2}}$ and aspect ratio (AR), defined as $\frac{\text{Major axis}}{\text{Minor axis}}$ (ImageJ User Guide, https://imagej.net/ij/docs/guide/index.html), were measured using Fiji software (ImageJ, v. 1.54f; Schindelin *et al*. 2012).

### Morphological analysis of flagellate cells by SEM

For detailed morphological characterisation of the flagellate cells by SEM, 1 mL of the BBM algal suspensions of each species (n = 20 per species), prepared as described above, were transferred to 2 mL Eppendorf tubes and centrifuged at 3500 rpm for 4 min to obtain pellets. The liquid BBM was removed, avoiding pellet resuspension, and replaced with ca. 1 mL of Karnovsky's fixative (2.5% paraformaldehyde, 0.5% glutaraldehyde in 0.1 M Sørensen's phosphate buffer). Fixation was carried out for 12 h at 4°C. The fixative was replaced twice with 1 mL of 0.1 M Sørensen's phosphate buffer which was left with the pellet for 5 min each time (2 × 5 min). Post‐fixation was then carried out by replacing the buffer with 1 mL of 2% OsO_4_ in 0.2 M phosphate buffer for 2 h at room temperature. The pellets were rinsed again with 0.1 M phosphate buffer (2 × 5 min) and dH_2_O (2 × 5 min) and then dehydrated by substituting dH_2_O with an ethanol (EtOH) graded series (30%, 50%, 70%, 95% and 100%), each step 2 × 10 min and the final of 3 × 15 min. The pellets were then resuspended, immobilised on cellulose acetate filter discs (25 mm diameter; 0.2 μm pore size; Sartorius Stedim Biotech, Göttingen, DE) using a vacuum filtering system and then dried completely using an Emitech K850 CO_2_ critical point dryer (Quorum Technologies, Lewes, UK). EtOH was removed stepwise by soaking the samples five times (10 min each) in liquid CO_2_; then the procedure was carried out according to the manufacturer's instructions. The use of filter discs resulted in very clear images, but also led to a significant loss of cells during the drying process. Therefore, a complementary method to decrease cell loss was used: some pellets were resuspended and placed in porous PTFE pots for critical point dryers (Quorum Technologies) then dried completely using the same procedure. Cellulose discs and cells were mounted on aluminium stubs coated with double‐sided carbon tape then carbon coated using the Q150T ES plus sputterer (Quorum Technologies, Lewes, UK) using the pulsed rod evaporation method. Samples were analysed with a SEM Gemini 300 (Zeiss); images were acquired by analysing both secondary electrons (acquired with the InLens Secondary Electron detector) and backscattered electrons (acquired with the Inlens Energy selective Backscatter detector and the annular Backscattered Detector), using an acceleration voltage of 5 kV and a working distance of 5 mm. The presence of osmium was confirmed with Energy Dispersive Spectroscopy performed using a XFlash 610 M probe (Bruker, Billerica, USA), with an acceleration voltage of 5 kV and a working distance of 8.5 mm.

### Staining and observation of flagellate cells by epifluorescence microscopy

To confirm the lipid nature of the vesicles in flagellate cells observed using SEM, the algal samples were stained with Nile red (Merck, Darmstadt, DE), which binds to lipids. Briefly, samples from algal suspensions of *T. decolorans* were prepared similarly to those for SEM, but fixation was performed with ca. 1 mL of 4% paraformaldehyde in 0.1 M Sørensen's phosphate buffer. After washing, the cells were incubated with 1 mL of 2 μg mL^−1^ Nile red in dimethyl sulfoxide for 1 h at room temperature (Huang *et al*. 2009). The Nile red was discarded, cells were washed as before and immediately analysed with epifluorescence microscopy using an Eclipse Ti (Nikon, Tokyo, JP).

### Molecular analyses

To investigate genomic evidence of possible sexual reproduction in the genus *Trebouxia*, a comprehensive dataset of 62 Trebouxiophyceae genome assemblies was built; this included six *Trebouxia* sp., one *T. gelatinosa* and one *T. decolorans* genomes. The *T. gelatinosa* transcriptome of Candotto Carniel *et al*. (2016) was also assembled from raw reads and added to the dataset. Reference sequences from nine established meiotic genes (i.e. dmc1, hop1, hop2, mer3, mnd1, msh4, msh5, rec8, spo11; Schurko & Logsdon 2008) in Trebouxiophyceae were obtained from Fučíková *et al*. (2015). Amino acid sequences of these genes were then used to obtain putative homologous sequences from the genomic resources. The pipeline used the following workflow: amino acid reference sequences were aligned to genomic sequences (and the transcriptome) using Exonerate (Slater & Birney 2005) protein2genome mode; the best hit for each gene was then picked according to similarity to the reference sequence (≥55%) and hit length. A second run of the pipeline was then launched adding to the reference sequences those obtained from the first run and relaxing the sequence similarity threshold to 40%.

The sequences obtained were then aligned using the codon‐aware MACSE2 method (Katoh *et al*. 2005; Ranwez *et al*. 2018) to further check the retrieved sequences. The identity of the sequences obtained from genomic resources was confirmed using BLASTn against the NCBI nucleotide database.

### Statistical analyses

Generalised linear models (GLM) were used to test for infraspecific variability in anatomical parameters (i.e. AR and circularity) of the flagellate cells of the four species using R software (R v. 4.3.2; R Core Team, 2023). GLMs were run, with each anatomical parameter as response variable, and ID of the microscope slide and cell shape as explanatory variables. The ID of the slide was used to verify if it significantly accounted for potential intra‐ and inter‐slide variability of the response variables. When testing for AR, GLMs were performed based on the gamma distribution using the glm function of the R package “stats”. To test for circularity, GLMs were performed on the basis of the beta distribution using the betareg function from the R package “betareg” (Cribari‐Neto & Zeileis 2010). Model assumptions of normality and homoscedasticity of residuals were visually checked using quantile‐quantile plots and residuals versus fitted values plots, respectively. In cases where the assumption of homoscedasticity was violated, a dispersion model for the cell shape was included in the beta regression models to account for the heteroscedasticity of the residuals.

## RESULTS

### Characterisation and development of zoospores

The algal cultures of all species produced zoospores between 18 and 22 days after subculture. The novel method used to observe the algal populations made it possible to follow the final stages of zoosporogenesis up to release of the zoospores and, subsequently, the developmental fate of the zoospores. The solid BBM layer on the microscope slides restricted distribution area of the zoospores, which was often limited to the optical field of the light microscope at the highest magnification (400×), without affecting zoospore mobility. In this way, many zoospores could be carefully observed without fixation and then characterised sequentially for a maximum of 14 days using images extracted from the recorded videos.

**Fig. 1:**
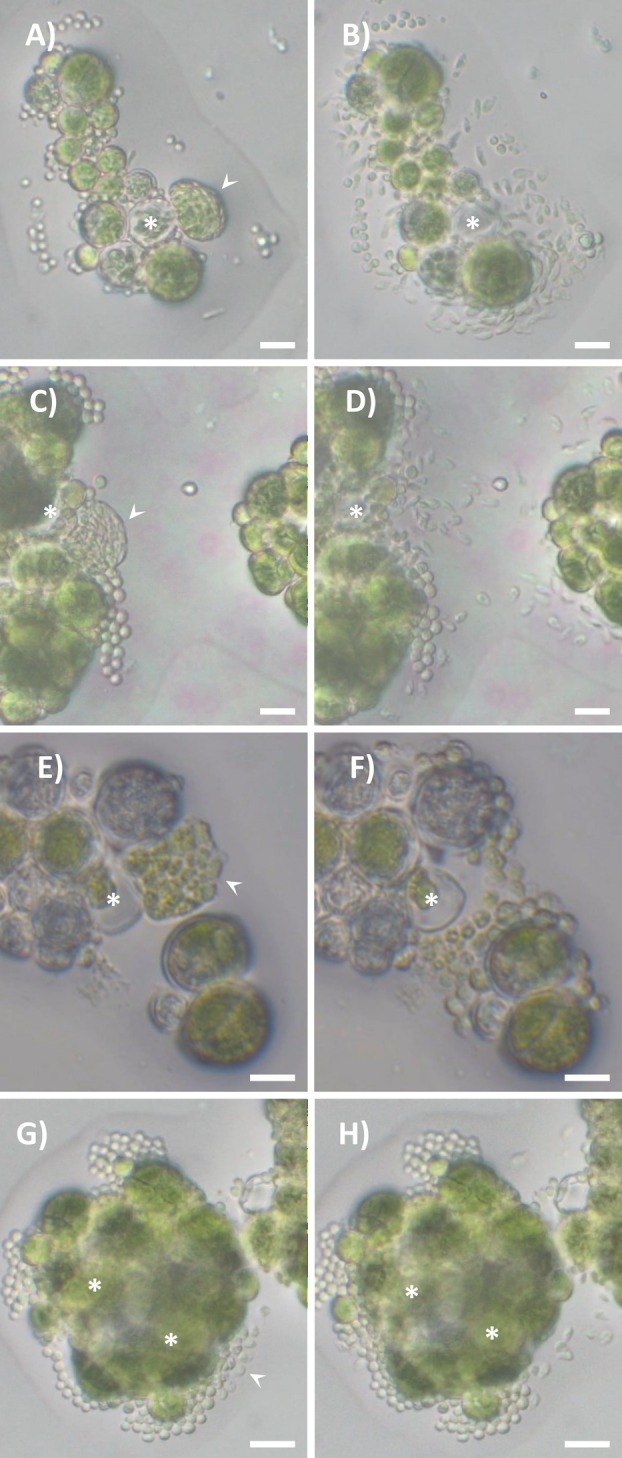
Release of zoospores in *Trebouxia decolorans* (A, B, E, F) and *T. vagua* (C, D, G, H) under light microscopy. Elongated (A–D) and subspherical (E–H) morphotypes. White asterisks indicate zoosporangia that release zoospores; white arrowheads indicate zoospores that are simultaneously released by zoosporangia. In each sequence, the time between first and last image is ca. 15 s. Scale bars = 5 μm.

**Fig. 2:**
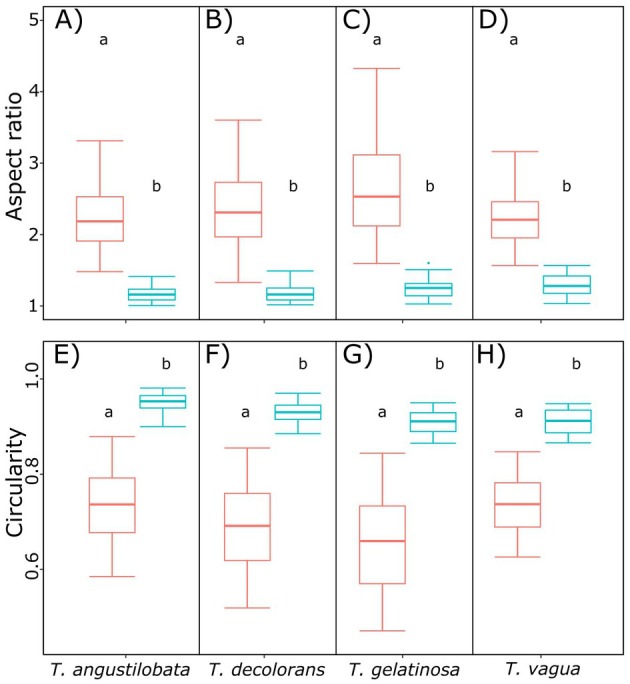
Boxplots of aspect ratio (A–D) and circularity (E–H) of zoospores of *Trebouxia angustilobata* (A, E; *n* = 163), *T. decolorans* (B, F; *n* = 231), *T. gelatinosa* (C, G; *n* = 173) and *T. vagua* (D, H; *n* = 163). Whiskers represent values lower or higher than the 25th and 75th percentile ±1.5 × interquartile range, respectively. Orange boxplots indicate elongated morphotype, light‐blue boxplots indicate subspherical morphotype. Different letters indicate statistically significant differences (glm, *p* < 0.05).

**Fig. 3:**
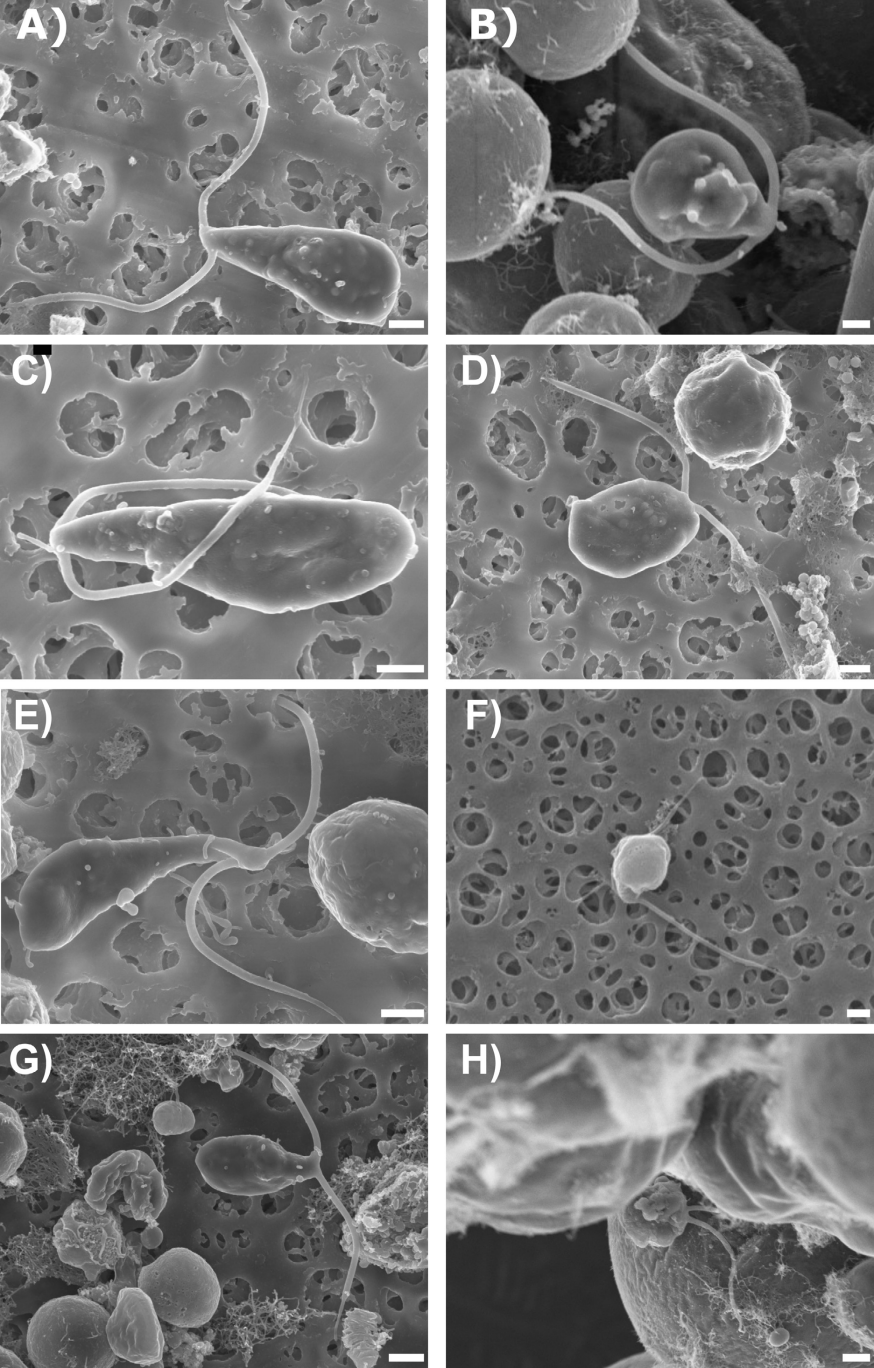
SEM photomicrographs of zoospores of *Trebouxia angustilobata* (A, B), *T. decolorans* (C, D), *T. gelatinosa* (E, F) and *T. vagua* (G, H), elongated (A, C, E, G) and subspherical (B, D, F, H) morphotype. Scale bars = 1 μm.

The mechanism of zoospore release was very similar in all species (Videos S1–, S4). In most cases, zoospores were released in dense clusters from the zoosporangia, surrounded by a gelatinous sheath, and all emerged through a crack in the cell wall of the sporangium (Fig. 1A,C,G); however, sometimes some zoospores failed to emerge from the mother cell and remained within its cell wall (Fig. 1E,F). Two types of zoospores could be distinguished in all four species: elongated and subspherical (Fig. 1). Their aspect ratio always differed statistically (Fig. 2A–D) and ranged from 1.8 to 3.2 in the elongated morphotype, and from 1.1 to 1.4 in the subspherical morphotype. Circularity also differed significantly between morphotypes in all species (Fig. 2E–H), ranging from 0.6 to 0.8 in the elongated morphotype and from 0.9 to 1.0 in the subspherical morphotype. Despite this morphological similarity, the zoospores of *T. vagua* were smaller than those of the other examined species (Fig. 3 and Table S1). The dimensions of the elongated flagellate cells were consistent with species descriptions of the protologues (Ahmadjian 1960; Archibald 1975; Beck 2002), with the exception of *T. vagua* (Voytsekhovich & Beck 2016) (Table S1). In all observations, only one morphotype was released from one and the same zoosporangium.

**Fig. 4:**
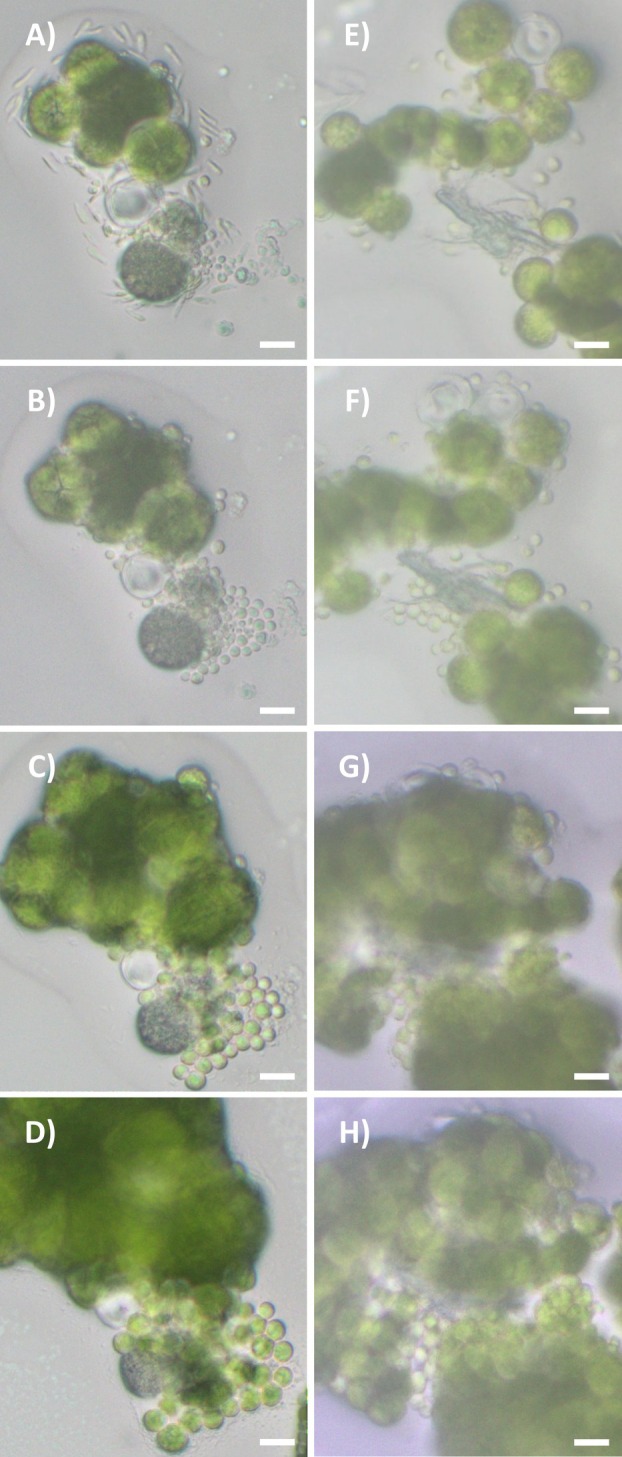
Zoospores of *Trebouxia decolorans* of the elongated (A–D) and subspherical (E–H) morphotypes observed under the light microscope at release (A, E), and after 1 (B, F), 4 (C, G), and 9 (D, H) days. Scale bars = 5 μm.

In all species, zoospores of both morphotypes stopped moving about 10 min after release and settled. (Fig. 4, Figs. S1 and S2). Within 24 h they became round vegetative cells, with a cell wall (Peveling & König 1985). In the following days, these cells increased in size until they began to divide around day 9 (Fig. 4, Figs. S1 and S2; no data available for subspherical zoospore of *T. gelatinosa* and *T*. *vagua*).

**Fig. 5:**
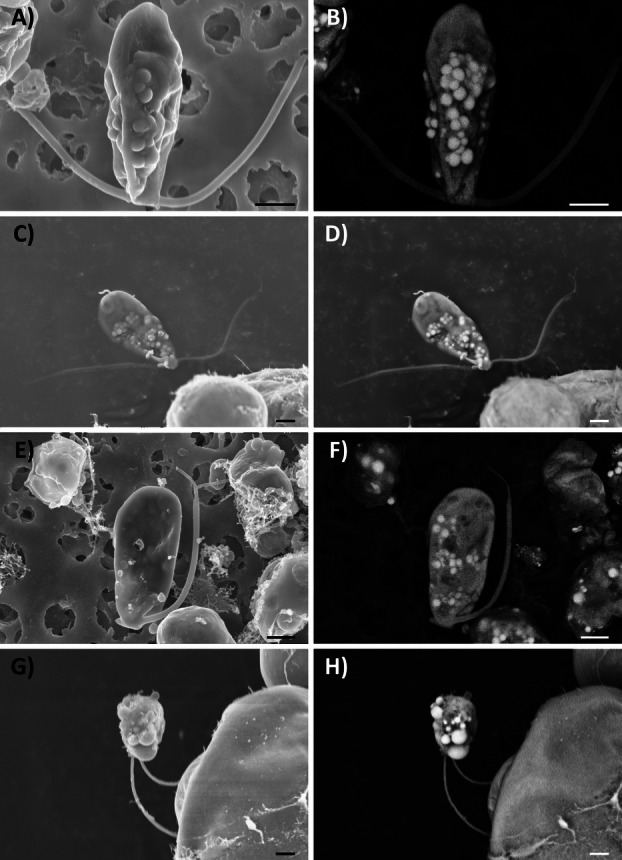
SEM photomicrographs of elongated flagellate cells of *Trebouxia angustilobata* (A, B), *T. decolorans* (C, D), *T. gelatinosa* (E, F), and subspherical flagellate cell of *T. vagua* (G, H) analysed with secondary (A, C, E, G) and backscattered (B, D, F, H) electrons. Bodies that appear bright due to the presence of osmium have been identified as lipid droplets. Scale bars = 1 μm.

The two morphotypes were also recognised as such in all investigated species using SEM (Fig. 3). Zoospores of both morphotypes, investigated by acquiring secondary electrons, appeared flattened, with two smooth anterior flagella of equal length, and a less regular surface than the surrounding vegetative cells. Zoospores of individual morphotypes appeared similar in all investigated species. Small hemispherical structures emerged on the surface, especially at the base of the flagellar apparatus and in the chloroplast region (e.g. Fig. 3A,D). When analysed with backscattered electrons to investigate the presence of a chemical contrast, these structures appeared very bright in contrast with other structures and the background, indicating the presence of heavyweight atoms; the Energy Dispersive Spectroscopy analysis confirmed the presence of osmium within these structures (Mαβ lines of osmium in the EDS spectrum; Fig. S3), indicating the presence of lipids, which are chemically bound to this element (Belazi *et al*. 2009; Bello *et al*. 2010; Teresa Núñez‐López *et al*. 2018), thus allowing identification of these structures as lipid bodies or droplets (Fig. 5). Staining of zoospores with Nile red allowed clear identification of lipid droplets outside the chloroplast. Although some photosynthetic autofluorescent pigments emitted at the same wavelength as Nile red, detection of the chlorophyll *a* autofluorescent signal allowed unambiguous identification of the stained lipid droplets and confirmed the SEM analysis (Fig. S4).

### Morphological evidence of cell fusion events

**Fig. 6:**
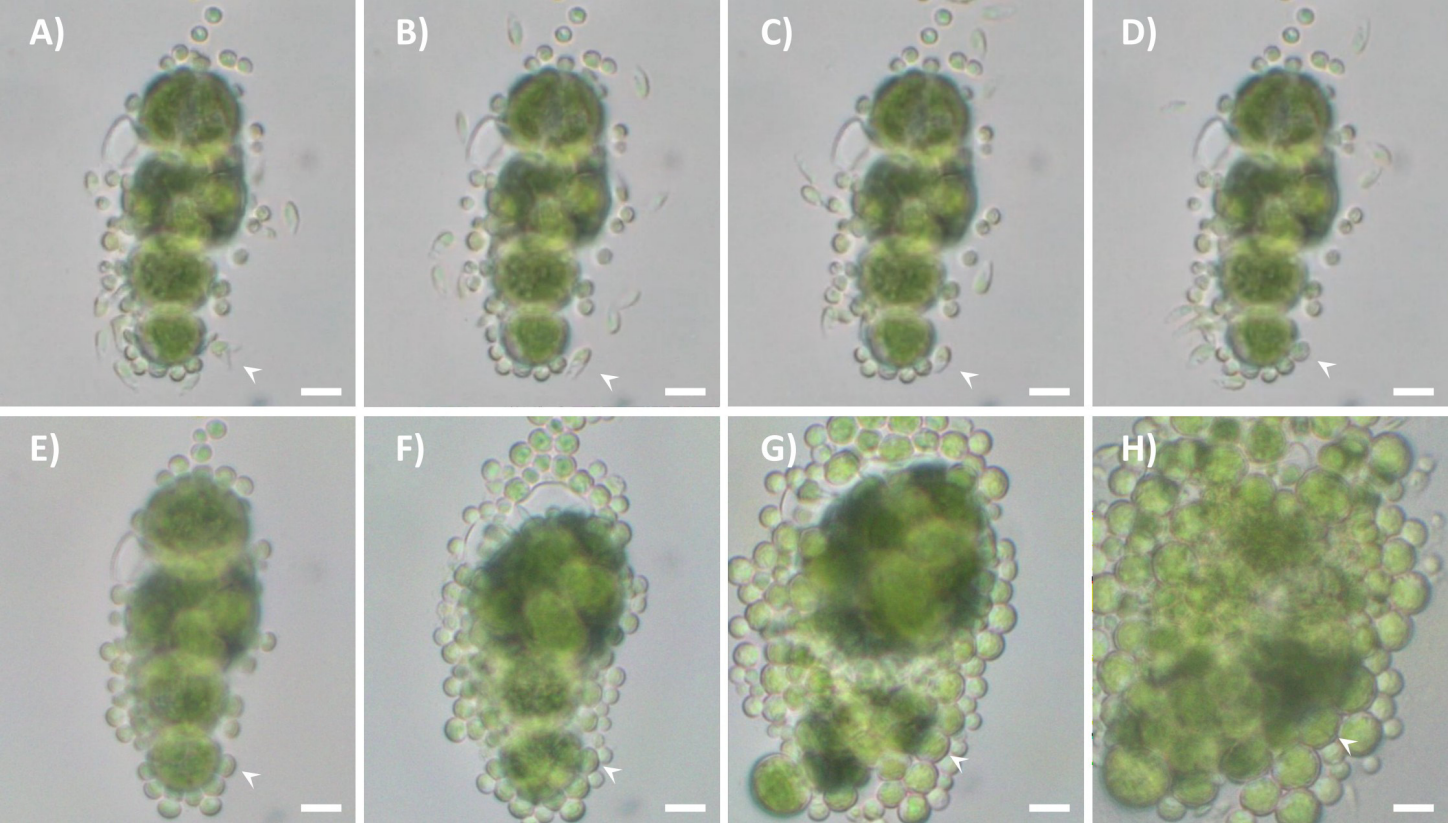
Algal cells of *Trebouxia angustilobata* under light microscopy. White arrowheads indicate two flagellate cells fusing and the following plasmogamic steps. This stage is interpreted as a sexual fusion. Flagellate cells progressively fusing via their posterior parts (A, B); formation of a cell with four flagella (C); subsequent four‐flagellate cell (D); four‐flagellate cell after 1 (E), 3 (F), 6 (G), and 10 (H) days. On day 10, the chloroplast started to divide into four parts (H). Scale bars = 5 μm.

After release, some of the elongated flagellate cells of *T. angustilobata* (Fig. 6, Videos S5–, S7) and *T. vagua* (Video S8) began to fuse. The fusion started at the posterior ends, with the flagella on the same side (Fig. 6 and Video S7) or in opposite directions (Videos S5, S6 and S8). When flagella were on the same side (Fig. 6A), fusion progressed along the major axis of the flagellate cells (Fig. 6B), eventually leading to formation of a single cell with four flagella (Fig. 6C), possibly a planozygote. Approximately 3 min after formation, this cell stopped moving and settled, losing both its elongated shape and its flagella (Fig. 6D). At this point it was identical in size and shape to the surrounding cells. After 10 days, the chloroplast of the putative planozygote divided into four parts (Fig. 6E–H). Further observations were not possible as this cell was accidentally covered by the surrounding vegetative cells so that it could no longer be recognised. The plasmogamic event between cells fusing in opposite directions was entirely the same as that described above for cells fusing with the flagellar apparatus in the same direction, but in those cases the putative zygotes could not be tracked in the following days (Videos S5, S6 and S8).

No fusions between subspherical cells or between elongated and subspherical flagellate cells were observed.

**Fig. 7:**
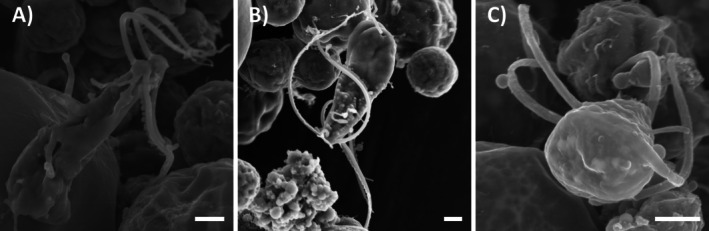
SEM photomicrographs of elongated flagellate cells of *Trebouxia angustilobata* undergoing cell fusion. Two flagellate cells fusing at their major axis (A); a cell with four flagella almost completely formed, with the anterior part completely fused and the posterior part almost fused (B); rear view of a four‐flagellate cell showing the complete fusion of the posterior parts (C). Scale bars = 1 μm.

The different stages of plasmogamic fusion between elongated flagellate cells of *T. angustilobata* were also observed using SEM (Fig. 7), and confirmed the events previously observed with the light microscope. However, the high magnification and quality of SEM examinations allowed the surface of the flagella of the fusing cells and the fusion of the cell surfaces during the plasmogamic process to be observed in more detail. In one stub, flagellate cells fused along their main axis, with the anterior parts separated and each cell retaining its own pair of flagella with ridge‐like structures on their surface (Fig. 7A). At a more advanced stage, the anterior parts completely fused into a single structure with four flagella, while the posterior parts were partially unfused, with a distinct furrow between the almost separate posterior parts; ridge‐like structures were still present on the flagella (Fig. 7B). Finally, a fully formed cell was observed in which the posterior part was completely fused (no furrow visible), indicating that plasmogamy was complete; no ridge‐like structures were observed on the flagella (Fig. 7C).

Further SEM observations revealed similar fusion events also in *T. decolorans* (Fig. S5).

### Molecular evidence of meiosis

All nine meiotic genes (i.e. dmc1, hop1, hop2, mer3, mnd1, msh4, msh5, rec8 and spo11; Schurko & Logsdon 2008; Fučíková *et al*. 2015) were detected in the Trebouxiophyceae genomes (Table S2). Highly conserved genes such as dmc1 and spo11 were consistently retrieved with nearly complete or complete hits (e.g. dmc1 relative hit length of ≥0.87) across all orders. In contrast, retrieval rate of the rec8 gene was strikingly low and often characterised by incomplete hits. Remarkably, in the genus *Trebouxia*, all meiotic genes were present with nearly complete hit length (0.87 average length) except for rec8, which was not detected. Furthermore, in the transcriptome of *T. gelatinosa*, seven out of nine genes were detected with a hit length of at least 0.70, with the exception of mer3, which was only partially retrieved (0.13 relative length), and rec8, which was not detected (Table 1).

**Table 1:** Heatmap showing the presence of nine meiotic genes (dmc1, hop1, hop2, mer3, mnd1, msh4, msh5, rec8, spo11; Schurko & Logsdon 2008, Fučíková *et al.* 2015) across different *Trebouxia* genomes and one *T. gelatinosa* transcriptome.

taxa	NCBI reference	DMC1	HOP1	HOP2	MER3	MND1	MSH4	MSH5	REC8	SPO11
*Trebouxia decolorans*	Unpublished data	**1.00**	**0.85**	**0.87**	**0.91**	**0.99**	**0.49**	**0.84**	**0.00**	**1.00**
*Trebouxia gelatinosa* (genome)	GCA_000818905.1	**0.93**	**0.85**	**0.00**	**0.00**	**1.00**	**0.70**	**0.00**	**0.00**	**0.91**
*Trebouxia gelatinosa* (transcriptome)	SRR988248	**0.97**	**0.86**	**0.94**	**0.13**	**1.00**	**0.70**	**1.00**	**0.00**	**0.94**
*Trebouxia* sp.	Unpublished data	**1.00**	**0.85**	**0.87**	**0.48**	**1.00**	**0.49**	**0.84**	**0.00**	**1.00**
*Trebouxia* sp.	GCA_008636185.1	**1.00**	**0.84**	**0.90**	**0.48**	**1.00**	**0.81**	**1.00**	**0.00**	**1.00**
*Trebouxia* sp.	GCA_040206735.1	**1.00**	**0.84**	**0.90**	**0.91**	**1.00**	**0.76**	**1.00**	**0.00**	**0.99**
*Trebouxia* sp.	GCA_040206755.1	**1.00**	**0.84**	**0.90**	**0.48**	**1.00**	**0.74**	**1.00**	**0.00**	**1.00**
*Trebouxia* sp.	GCA_040206745.1	**1.00**	**0.84**	**0.00**	**0.48**	**1.00**	**0.78**	**1.00**	**0.00**	**0.99**
*Trebouxia* sp.	GCA_002118135.1	**1.00**	**0.85**	**0.99**	**0.91**	**1.00**	**0.78**	**1.00**	**0.00**	**1.00**

Numerical values in bold represent relative length of the obtained sequences in comparison to reference sequences.

## DISCUSSION

Despite the crucial role of flagellate cells in the life cycle of many Chlorophyta, little attention has been paid to the motile stages of *Trebouxia* compared to the non‐motile vegetative cells. The latter have the most important taxonomic traits, such as type of cell division, chloroplast morphology, and pyrenoid ultrastructure, which have been the main focus of taxonomic research to date (e.g. Peveling & König 1985; Friedl 1989; Bordenave *et al*. 2022). On one hand, observations of potential gametic fusions have been sporadic, with few reports in the literature (Warén 1920; Jaag 1929; Ahmadjian 1960, 1967; Komárek & Fott 1983), and molecular analyses indicating the possibility of sexual reproduction also being limited (Kroken & Taylor 2000; Gazquez *et al*. 2024). On the other hand, it has been proposed that all flagellate cells in *T. lynniae* are gametes, with no evidence of the presence of zoospores (Gazquez *et al*. 2024). As a result, the nature of flagellate cells in the life cycle of *Trebouxia* remains ambiguous and further investigations are clearly needed.

### Characterisation and development of zoospores

Videos of the release of zoospores in *Trebouxia* confirmed that they were closely packed with a gelatinous sheath during release from the sporangium in all species studied here (Fig. 1, Videos S1–, S4), as previously described not only for some *Trebouxia* species (e.g. Famintzin & Baranietzky 1867), but also for the closely related genus *Asterochloris* (Škaloud *et al*. 2015). Shortly after release from the sporangia, two zoospore morphotypes, that is, elongated and subspherical, could be distinguished, which differed significantly both in aspect ratio and circularity (Fig. 2A–D and E–H, respectively). The different morphotypes were released from different sporangia (Fig 4, Figs. S1 and S2, Videos S1–, S4). Zoospores in *Trebouxia* (Archibald 1975; Tschermak‐Woess 1989), as well as in other algal species such as *Neochloris aquatica* (Starr 1955), typically lose their elongated shape when motility decreases shortly after release, become spherical, lose their flagella, and start cell wall development (Peveling & König 1985). This may have led to interpretation of the subspherical zoospores as elongated zoospores that settled. Real‐time monitoring allowed us to definitively distinguish between the two morphotypes, as the subspherical flagellate cells were released from different sporangia than the elongated cells, suggesting that the former are not just a development stage of the latter. Moreover, the elongated flagellate cells generally lost motility when they rounded, whereas the subspherical cells were as motile as the elongated cells. In the Chlorophycean *Chlorosarcinopsis gelatinosa*, Melkonian *et al*. (1980) showed that temperature changes (cold treatment at 2°C for 4–5 min) can induce shape changes in zoospores, but since in our study both morphotypes consistently co‐occurred under the same environmental conditions, we conclude that dimorphism in *Trebouxia* is not related to environmental factors.

Flagellate cells of the subspherical morphotype were less common than elongated cells in all examined species, suggesting that they are rarer (Table S1). Jaag (1929: pp. 90–97) reported the presence of two distinct zoospore morphotypes, elongated and subspherical, in *Trebouxia* sp. (*sub Cystococcus parmeliae*) from the lichen *Flavoparmelia caperata*. This first observation of the diversity of *Trebouxia* zoospores suggested possible different functions among morphotypes. Notably, these findings were largely overlooked in later studies, resulting in the lack of a description of zoospore dimorphism in *Trebouxia* species until now.

The sedimentation time of both morphotypes and their development (i.e. from cell wall production 24 h after release to cell division after 9 days) are in perfect agreement with earlier reports on the development of vegetative cells from zoospores in other *Trebouxia* species under similar culture conditions (Woronine 1872; Tschermak‐Woess 1989; Friedl 1993). Based on these observations, we conclude that the two morphotypes of flagellate cells are indeed zoospores and that it may be necessary to update existing species descriptions to account for this dimorphism, the origin and significance of which remain unknown. We originally hypothesised that the different morphotypes might indicate anisogamy. However, this would have required the presence of mating type regions in the genomes of *Trebouxia*. We were able to identify some mating‐type genes from Ulvophyceae and Chlorophyceae in the *Trebouxia* genomes, but the alignments were too short to confidently infer homology. Furthermore, our observations indicate that both morphotypes undergo vegetative development and are thus zoospores. The observation of these morphotypes in species belonging to three different clades of *Trebouxia* (clades S, A and I; clade nomenclature according to Xu *et al*. 2020) suggests that this feature is conserved throughout the genus. Given the potential role of this dimorphism in the reproductive diversity of *Trebouxia*, further research should investigate: (i) whether it is also present in the remaining clades C and D of *Trebouxia* to gain a more complete understanding of its significance within the genus; (ii) possible differences in the organisation or function of the cytoskeleton, because these factors may lead to variation in the cell shape of the two morphotypes (Melkonian *et al*. 1980).

### Morphological evidence of cell fusion events

Our study provides direct evidence for cell fusion events in three of the four species analysed. Video recordings of flagellate cells fusion in *Trebouxia* were obtained for the first time, making it possible to observe all steps of the plasmogamic process in detail. We observed fusion events only between elongated cells and never between subspherical cells. In all cases, the fusion site was located in the posterior part of the elongated cells, close to the chloroplast and the nuclear region (Fig. 6, Videos S5–, S8), but the flagellate cells could approach each other with the flagellar apparatus pointing in the same direction or in opposite directions. These observations confirm the illustrations of Warén (1920) and Ahmadjian (1960). Jaag (1929) reported anisogamy in *Trebouxia*, but we were unable to observe fusion between elongated and subspherical flagellate cells. In our system, the algal cells deposited on the solid BBM layer formed a “water pocket” in which the flagellate cells were confined. In most cases, there was only one zoosporangium releasing flagellate cells (Fig. 1A–F) of the same morphotype. Sometimes, however, more zoosporangia were present in the same cell group (Fig. 1G,H). Very rarely, two zoosporangia close to each other released two different morphotypes of flagellate cells; but in these cases, we never observed fusion events between the two morphotypes. Interestingly, Gazquez *et al*. (2024), who observed only subspherical flagellate cells in *T. lynniae*, stated that in this species all flagellate cells are gametes, with no evidence of the presence of zoospores. Based on our observations, their findings cannot be extended to all Trebouxias, and hence it is difficult to accept the proposal (of these authors) that *T. lynniae* is “the” model species for the whole genus *Trebouxia*. Meanwhile we must try to explain why *T. lynniae* apparently produces only gametes. One possible explanation could lie in the culture conditions, as these authors used a growth medium containing casein hydrolysate as a source of organic nitrogen, instead of casein peptone of the regular *Trebouxia* medium (Ahmadjian 1973). It is known that nitrogen availability strongly influences the cell cycle of green algae, such as *Chlamydomonas* (Žárský *et al*. 1985). Possibly, the same holds true for *Trebouxia*. Incidentally, Gazquez *et al*. (2024) did not clarify what happens to the gametes of *T. lynniae* that do not fuse. Hence, it would be interesting to apply our method to follow their development and thus confirm that none undergo vegetative development (i.e. none are zoospores).

The novel method we used allowed us to obtain unprecedented LM images of development of a cell formed by fusion of flagellate cells. In Video S7 and the relative images in Fig. 6 of *T. angustilobata*, the zygote appears to have only one chloroplast a few minutes after the plasmogamic event. This seems to be a general rule in green algae (for a review, see Miyamura 2010), where the single chloroplast of the zygote might result from disintegration of one of the two chloroplasts (Friedmann 1960; Bråten 1971) or from fusion of the two chloroplasts (Crawley 1966; Brown *et al*. 1968). It is noteworthy that Ahmadjian (1960) observed, in a single case, the fusion of gametes in *T. impressa*, but that the zygote retained two distinct chloroplasts that split into two after a few days. This could indicate that the presence of a single chloroplast in the zygote is not consistent in all *Trebouxia* species.

Ten days after the plasmogamic event documented in Video S7, the chloroplast of the putative zygote started to divide into four parts (Fig. 6H). This could be a phase of a meiotic event leading to the formation of four vegetative cells, as described for *Chlamydomonas* (Sekimoto 2017). The SEM images of the plasmogamic events provided useful information that would have been very difficult to obtain with LM. The different stages of the fusion process in *Trebouxia* showed some similarities to sexual reproduction in other green algae. In the isogamous *Chlamydomonas*, sexual reproduction begins with recognition between the gametes through interaction of their flagella (Žárský *et al*. 1985). To facilitate flagella adhesion, agglutinins are produced on their surface, forming “fringe” structures that also trigger mating structure activation and cell membrane fusion (Adair 1985). Interaction between flagellar agglutinins brings gametes sufficiently close to facilitate subsequent cell fusion (Mori *et al*. 2015). After adhesion and the onset of cell fusion, agglutinins are inactivated and are no longer found on the surface of flagella (Van den Ende *et al*. 1988). Similarly, in *T. angustilobata*, as the elongated flagellate cells began to align and fuse, ridge‐like structures appeared on membranes of the adherent flagella; at a later stage, the ridge‐like structures were still present, but flagella were no longer attached to each other; when the planozygote was fully formed, no structures were present on the flagella (Fig. 7). Accordingly, the fully formed planozygotes of *T. decolorans* also lacked structures on the flagella (Fig. S5).

Based on these observations and their similarity to the steps of sexual reproduction in other green algae, we conclude that the flagellate cells described in this section are sexual gametes. Putative gametes have always been described as indistinguishable from zoospores (Warén 1920; Jaag 1929; Ahmadjian 1960, 1967; Komárek & Fott 1983), but according to our SEM images, flagellar morphology seems to be a key character to distinguish between the two flagellate life stages.

### Molecular evidence of meiosis

To further confirm the presence of sexual reproduction in *Trebouxia*, as suggested by morphological observations using LM and SEM, we searched for the presence of established meiotic genes in a comprehensive dataset of 62 assembled genomes of Trebouxiophyceae (including six *Trebouxia* sp., one *T. gelatinosa*, and one *T. decolorans*) and one transcriptome (*T. gelatinosa*; Candotto Carniel *et al*. 2016). Since meiosis is a process that is essential for sexual reproduction, these genes can be used to detect the latter in organisms from different kingdoms (Schurko & Logsdon 2008). Some of these genes have even been detected in species of the Trebouxiophyceae in which sexual reproduction has never been observed (Blanc *et al*. 2010; Fučíková *et al*. 2015), leading to the suggestion that cryptic sexuality exists in several taxa of this class (Friedl & Rybalka 2012). However, for *Trebouxia*, information on the presence of meiotic genes is only currently available for *T. lynniae* (Gazquez *et al*. 2024). The confirmation that in all *Trebouxia* species analysed almost all had target genes reinforces the assumption that sexual reproduction is present in the genus (Table 1). The absence of hop2, mer3, and msh5 in the genome of *T. gelatinosa* (NCBI reference: GCA_000818905.1) and of hop2 in *Trebouxia* sp. (NCBI reference: GCA_040206745.1) is most likely due to incomplete draft assemblies, rather than actual absence from the genome, as the same genes were found in all other Trebouxias. Interestingly, the retrieval of rec8 varied even among species of the same genus and was extremely low overall (Table S2). rec8 is a meiosis‐specific paralog of rad21, which is one of the genes that form cohesin, a multi‐subunit complex that regulates cohesion of sister chromatids (Schurko & Logsdon 2008). Given the presence of the other searched meiosis gene, the absence of this gene in many Trebouxiophyceae genomes is possibly due to its higher sequence variability, as highlighted by the difficulty in alignment of the few obtained sequences of this gene (data not shown). However, the absence of a single gene related to meiosis is not an indication that meiosis cannot occur in these taxa (Schurko & Logsdon 2008).

Through the analysis described here, extended also to the transcriptome of *T. gelatinosa*, we have established that the algal populations actively express most of the meiotic genes during cell development. Certainly, further analyses, such as functional analyses of these genes, should be performed to confirm that they maintain their function and indeed play a role in sexual reproduction. However, this molecular evidence, combined with the morphological observations using LM and SEM, support the presence of sexual reproduction in *Trebouxia*, although it is a rare event, at least in the species and conditions used here. Further confirmations derive from observations of gamete fusion occasionally reported in the past (Warén 1920; Jaag 1929; Ahmadjian 1960, 1967; Komárek & Fott 1983), which confute the assumption that *Trebouxia* does not undergo sexual reproduction (Gärtner 1985; Friedl & Büdel 2008).

### The nature of *Trebouxia* flagellate cells

The nature of the elongated flagellate cells of the *Trebouxia* genus must be clarified. Two hypotheses can be made. In the first, these cells are either gametes or zoospores originating from different gametangia or zoosporangia, and gametes that do not undergo plasmogamy develop vegetatively, as observed in other green algae, such as *Chlamydomonas* (Van Den Ende 1994). This would mean that there are three different types of flagellate cells: gametes (elongated) and two zoospore types (elongated or subspherical). In the second hypothesis, the elongated flagellate cells are exclusively gametes that can still develop vegetatively, whereas the subspherical cells are zoospores. The distinction between “zoospore” and “gamete” becomes particularly blurred if *Trebouxia* is a haploid organism (like many closely related green algae), since both the gametes capable of vegetative development and the zoospores are haploid and arise from mitotic divisions. However, if *Trebouxia* is diploid, as proposed by Gazquez *et al*. (2024) for *T. lynniae*, the terms “zoospore” and “gamete” would denote different life stages, with the diploid zoospores arising from mitotic divisions and the haploid gametes from meiotic divisions. Nevertheless, the fact that no haploid vegetative stages originating from non‐fused gametes were observed in the abovementioned study raises further questions. At the present stage of our knowledge, we cannot decide in favour of one or the other hypothesis. Targeted studies focusing on the ploidy of the flagellate cells and their development are required to determine whether the elongated flagellate cells can only be gametes or also zoospores *sensu stricto*.

### Innovative methodologies for studying *Trebouxia* flagellate cells

The method presented here proved to be extremely effective for observing and analysing *Trebouxia* flagellate cells. Inoculation of the algae on a solid BBM layer on microscope slides not only allowed detailed characterisation of cell development and interactions within confined cell groups, providing unprecedented insight into dynamic cellular processes, but also real‐time observation of flagellate cells from their release up to 14 days. This is an improvement to conventional microscopy techniques that often rely on fixed cells. With this simple, extremely cheap setup, videos of the release of zoospores were recorded for the first time, capturing events that have previously only be inferred from illustrations (Famintzin & Baranietzky 1867; Jaag 1929; Gärtner 1985; Tschermak‐Woess 1989). In addition, we were able to clearly demonstrate sexual reproduction by documenting fusion of the gametes and the formation and subsequent development of a zygote using images and videos. Use of SEM allowed characterisation of zoospores at an unprecedented level of detail of their morphology (Fig. 3). The acceleration voltage of 5 kV allowed electrons to penetrate the cell membrane to the extent that some small, hemispherical structures became visible, especially below the region of the flagellar apparatus and in the area of the chloroplast (e.g. Figs 3A,D and 5). Analyses collecting backscattered electrons (whose emission is proportional to atomic weight of the atoms of the sample) showed that the hemispherical structures are very bright compared to other biological structures, suggesting that they are rich in lipids, affine to the osmium used for staining. This is consistent with previous studies in which OsO_4_ was used to stain lipid vesicles in TEM analysis (Bello *et al*. 2010), but also to stain biological samples in block face scanning electron microscopy (BFSEM) imaging, to obtain TEM‐like images (Núñez‐López *et al*. 2018; Mukhamadiyarov *et al*. 2021; Antreich *et al*. 2024). In the present work strategies were combined: OsO_4_ was successfully used to stain the zoospores, which were analysed using the backscattered electrons emitted by the osmium as in BFSEM but without sectioning the samples (as in BFSEM or TEM). The electron beam energy was in fact low enough to achieve high resolution but sufficient to penetrate the zoospores, thus allowing analysis of both the morphology of the zoospores and the presence of the lipid vesicles, as further confirmed by Nile red staining (Fig. 5, Figs. S3 and S4). One of the great advantages of SEM techniques is the fast and easier sample preparation compared to TEM. In a single study, zoospores were visualised using low temperature SEM (LTSEM) (Bordenave *et al*. 2023), but this technique, according to the authors, caused frequent cytolysis of the zoospores, which must be avoided for accurate morphological investigations. Consequently, frontal visualisation of superficial cell structures in zoospores is much easier with conventional SEM techniques compared to TEM and LTSEM.

## CONCLUSIONS

In this study, we investigated motile stages of four *Trebouxia* species using a novel method that enabled real‐time observations using LM techniques on the life span of single cells or cell groups. We captured unprecedented videos of zoospore release and fusion of flagellate cells, including the formation and development of putative zygotes. Our results confirmed the presence of two distinct morphotypes of flagellate cells, as well as evidence of gamete fusion, an important step in sexual reproduction. These morphological observations were further supported by molecular analyses, which confirmed the presence and expression of essential meiotic genes. We therefore conclude that there are two types of flagellate cells in the four *Trebouxia* species analysed: zoospores, which participate in asexual reproduction, and gametes, which are instead involved in sexual reproduction. Furthermore, the different morphology of the zoospores does not underlie a different function, but their origin remains unclear. Overall, this study provides convincing evidence for the occurrence of sexual reproduction in *Trebouxia* and represents a significant advance in understanding of the life cycle of this important genus of lichen‐forming green algae.

## AUTHOR CONTRIBUTIONS

EB: data curation, formal analysis, investigation, methodology, validation, visualisation, writing—original draft preparation; DP: formal analysis, investigation, methodology, resources, writing—review and editing; CGA: data curation, formal analysis, investigation, methodology, writing—review and editing; FCC: conceptualization, resources, supervision, visualisation, writing—review and editing; MT: conceptualization, project administration, resources, supervision, writing—review and editing.

## FUNDING INFORMATION

This work was supported by the University of Trieste [DOTTAMBIENTEVITA37‐BOCCATO‐22] and [D40‐microgrants22_CANDOTTO].

## CONFLICT OF INTEREST

No conflict of interest declared.

## Supporting information


**Fig. S1.** Zoospores of the elongated morphotype of *Trebouxia angustilobata* (A–D), *T. gelatinosa* (E–H), and *T. vagua* (I–L) observed under light microscopy at release (A, E, I), and after 1 (B, F, J), 4 (C, G, K), and 9 (D, H, L) days. Scale bars = 5 μm.
**Fig. S2**. Zoospores of the subspherical morphotype of *Trebouxia angustilobata* observed under light microscopy at release (A), and after 1 (B), 4 (C), and 9 (D) days. Scale bars = 5 μm.
**Fig. S3**. Representative Energy Dispersive Spectroscopy analysis performed on elongated flagellate cells post‐fixed with OsO_4_ characterised by subspherical bodies. The spectrum of osmium Mab lines shows presence of osmium in the bodies, which have been identified as lipid droplets.
**Fig. S4**. Subspherical flagellate cells of *Trebouxia decolorans* stained (+) or unstained (−) with Nile red, observed under epifluorescence microscopy in brightfield (A, E), with filters at 585 (B, F) and 680 (C, G) nm and in a merged composite (D, H). Lipid droplets of stained cells located outside the chloroplast (compare Fig. S4B with Fig. S4C) emit a vivid 585 nm (yellow) fluorescence (B, arrowheads). At the same wavelength the unstained cells emit only a weak yellow fluorescence signal (F) that matches the Chl*a*F signal (G), possibly due to some autofluorescent accessory pigments of the chloroplast. Scale bars = 5 μm.
**Fig. S5**. SEM photomicrographs of two planozygotes (A, B) of *Trebouxia decolorans* after plasmogamic events. Scale bars = 1 μm.
**Table S1**. Major and minor axes, aspect ratio and circularity of the two morphotypes (E: elongated; S: subspherical) of zoospores in four species of *Trebouxia*. Values are means ± SD. (n): number of cells used for these measurements.
**Table S2**. Presence of nine meiotic genes (i.e., dmc1, hop1, hop2, mer3, mnd1, msh4, msh5, rec8, spo11; Schurko & Logsdon 2008; Fučíková et al. 2015) across 62 assembled Trebouxiophyceae genomes and one *Trebouxia gelatinosa* transcriptome. Numerical values represent relative length of obtained sequence compared to reference sequence. Data referring to *Trebouxia* species are in bold.

**Video S1.** Release of elongated zoospores in *Trebouxia decolorans* observed under light microscopy. Video related to Fig. 1 (A, B).

**Video S2.** Release of elongated zoospores in *Trebouxia vagua* observed under light microscopy. Video related to Fig. 1 (C, D).

**Video S3.** Release of subspherical zoospores in *Trebouxia decolorans* observed under light microscopy. Video related to Fig. 1 (E, F).

**Video S4.** Release of subspherical zoospores in *Trebouxia vagua* observed under light microscopy. Video related to Fig. 1 (G, H).

**Video S5.** Plasmogamic event between gametes in *Trebouxia angustilobata*, with fusion started at posterior ends, and flagella in opposite directions.

**Video S6.** Plasmogamic event between gametes in *Trebouxia angustilobata*, with fusion started at posterior ends, and flagella in opposite directions.

**Video S7.** Plasmogamic event between gametes in *Trebouxia angustilobata*, with fusion started at posterior ends, and flagella on the same side. Video related to Fig. 6 (A–D).

**Video S8.** Plasmogamic event between gametes in *Trebouxia vagua*, with fusion started at posterior ends, and flagella in opposite directions.

## Data Availability

The data that support the findings of this study are openly available in “Mendeley Data” at https://data.mendeley.com/datasets/mdnz77znf5/1.
