# Supplementary material for: Zoospore diversity and sexual reproduction in the lichen‐forming genus Trebouxia: From neglected evidence to new facts
Source: Plant Biol (Stuttg). 2025 Jun 3;27(6):1137–49. doi: 10.1111/plb.70042 (PMC12477308; doi:10.1111/plb.70042)
Supplement: Supplementary file 1 — Fig. S1. Zoospores of the elongated morphotype of Trebouxia angustilobata (A–D), T. gelatinosa (E–H), and T. vagua (I–L) observed under light microscopy at release (A, E, I), and after 1 (B, F, J), 4 (C, G, K), and 9 (D, H, L) days. Scale bars = 5 μm. Fig. S2. Zoospores of the subspherical morphotype of Trebouxia angustilobata observed under light microscopy at release (A), and after 1 (B), 4 (C), and 9 (D) days. Scale bars = 5 μm. Fig. S3. Representative Energy Dispersive Spectroscopy analysis performed on elongated flagellate cells post‐fixed with OsO4 characterised by subspherical bodies. The spectrum of osmium Mab lines shows presence of osmium in the bodies, which have been identified as lipid droplets. Fig. S4. Subspherical flagellate cells of Trebouxia decolorans stained (+) or unstained (−) with Nile red, observed under epifluorescence microscopy in brightfield (A, E), with filters at 585 (B, F) and 680 (C, G) nm and in a merged composite (D, H). Lipid droplets of stained cells located outside the chloroplast (compare Fig. S4B with Fig. S4C) emit a vivid 585 nm (yellow) fluorescence (B, arrowheads). At the same wavelength the unstained cells emit only a weak yellow fluorescence signal (F) that matches the ChlaF signal (G), possibly due to some autofluorescent accessory pigments of the chloroplast. Scale bars = 5 μm. Fig. S5. SEM photomicrographs of two planozygotes (A, B) of Trebouxia decolorans after plasmogamic events. Scale bars = 1 μm. Table S1. Major and minor axes, aspect ratio and circularity of the two morphotypes (E: elongated; S: subspherical) of zoospores in four species of Trebouxia. Values are means ± SD. (n): number of cells used for these measurements. Table S2. Presence of nine meiotic genes (i.e., dmc1, hop1, hop2, mer3, mnd1, msh4, msh5, rec8, spo11; Schurko & Logsdon 2008; Fučíková et al. 2015) across 62 assembled Trebouxiophyceae genomes and one Trebouxia gelatinosa transcriptome. Numerical values represent relative length of obtained seq [file PLB-27-1137-s009.docx]

**
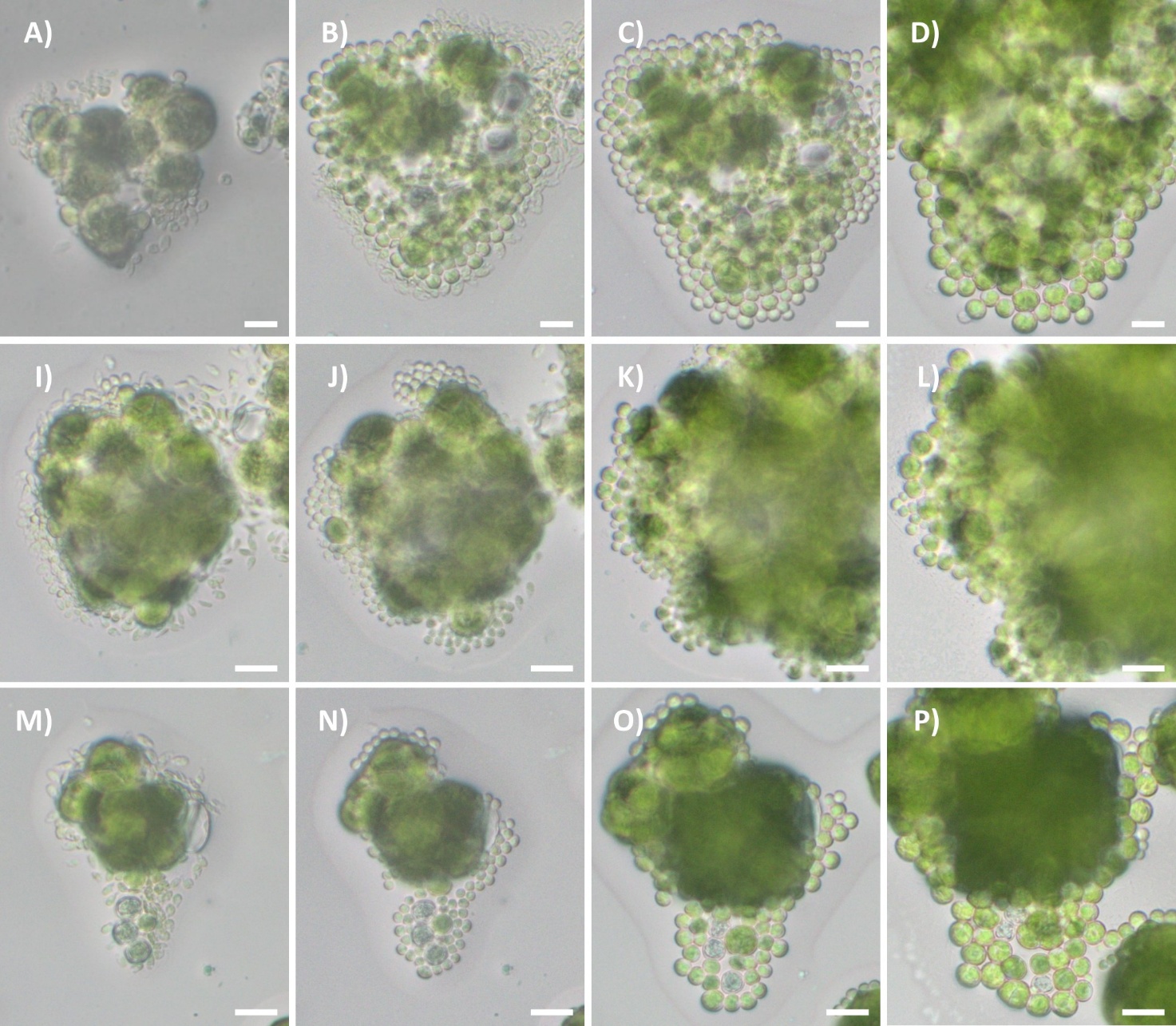
**

**Fig. S1.** Zoospores of the elongate morphotype of *Trebouxia angustilobata* (A-D), *T. gelatinosa* (E-H), and *T. vagua* (I-L) observed at light microscopy at release (A, E, I), and after one (B, F, J), four (C, G, K), and nine (D, H, L) days. Scale bars = 5 μm.


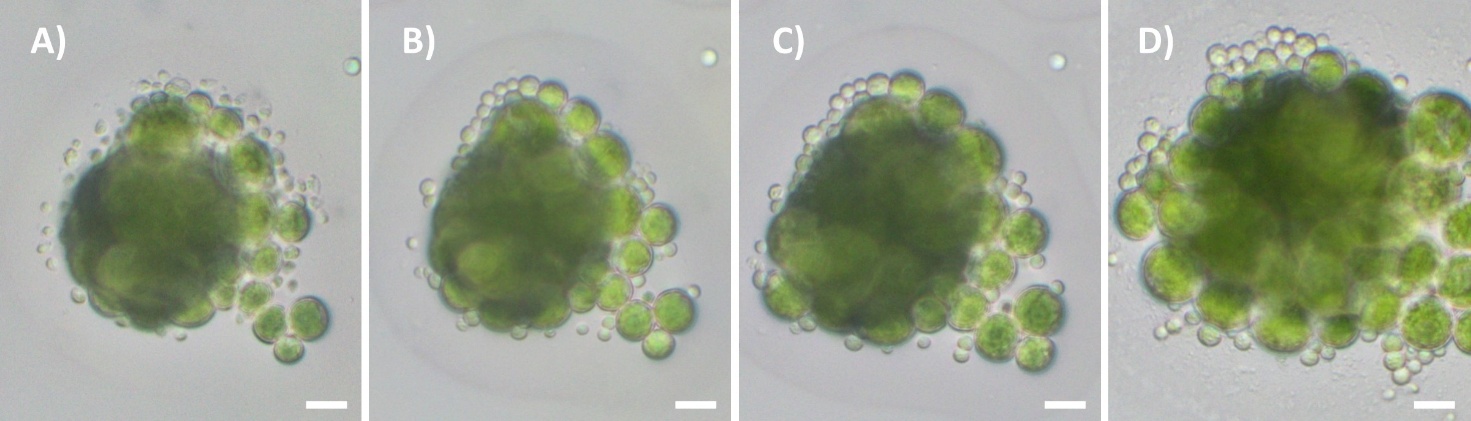


**Fig. S2.** Zoospores of the subspherical morphotype of *Trebouxia angustilobata* observed at light microscopy at release (A), and after one (B), four (C), and nine (D) days. Scale bars = 5 μm.


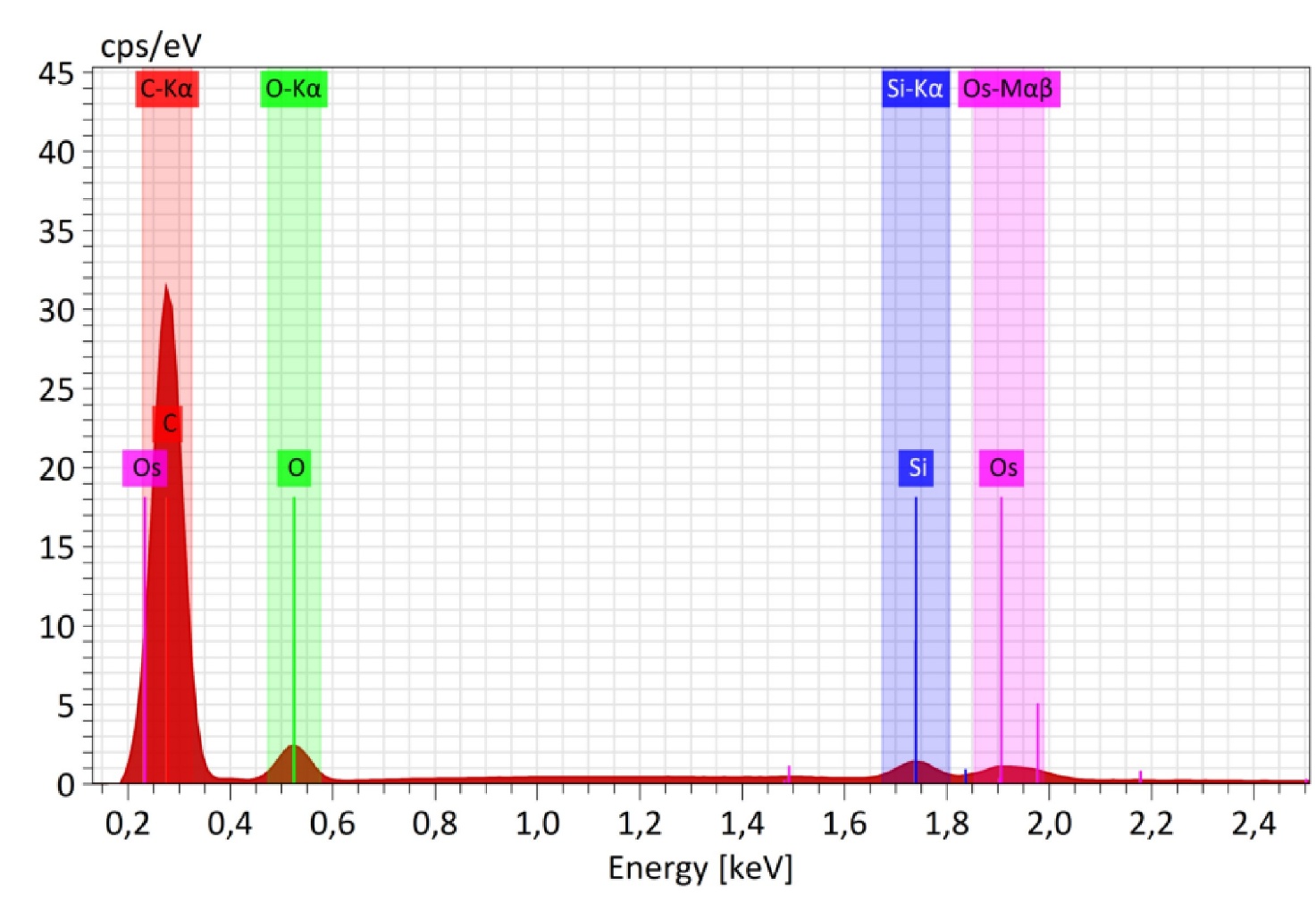


**Fig S3.** Representative Energy Dispersive Spectroscopy analysis performed on elongate flagellate cells post-fixed with OsO_4_ characterised by subspherical bodies The spectrum of osmium Mab lines shows the presence of osmium in the bodies, that have been identified as adiposomes.


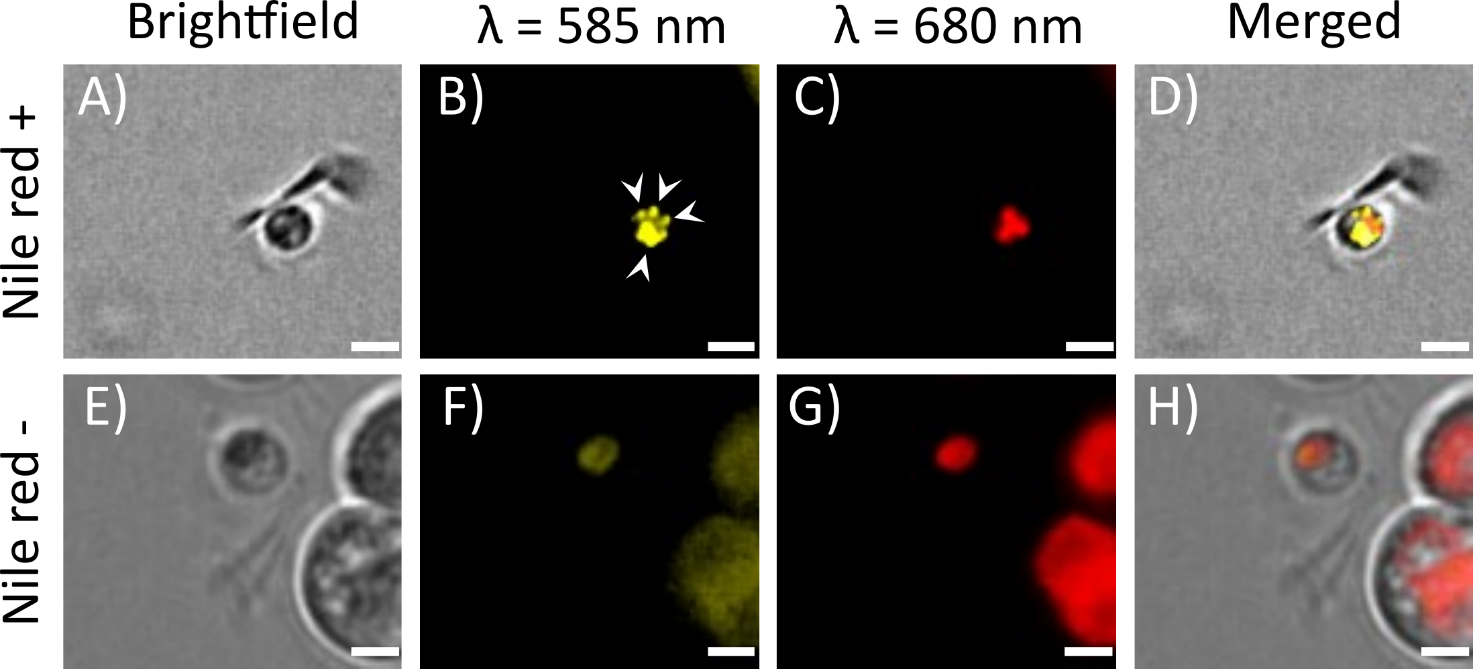


**Fig S4.** Subspherical flagellate cells of *Trebouxia decolorans* stained (+) or unstained (-) with Nile red observed at the epifluorescence microscope in brightfield (A, E), with filters at 585 (B, F) and 680 (C, G) nm and in a merged composite (D, H). Lipid droplets of stained cells located outside the chloroplast (compare Fig. S4B with Fig. S4C) emit a vivid 585 nm (yellow) fluorescence (B, arrowheads). At the same wavelength the unstained cells emit only a weak yellow fluorescence signal (F) that matches the Chl*a*F signal (G), possibly due to some autofluorescent accessory pigments of the chloroplast. Scale bars = 5 μm.


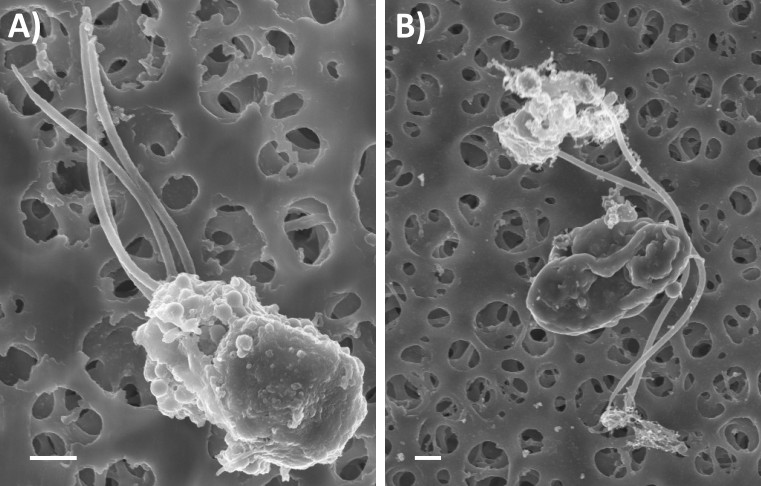


**Fig. S5.** SEM photomicrographs of two planozygotes (A, B) of *Trebouxia decolorans* after plasmogamic events. Scale bars = 1 μm.

**Tab. S1.** Major and minor axes, aspect ratio and circularity of the two morphotypes (E: elongate; S: subspherical) of zoospores in four species of *Trebouxia*. Values are reported as means with ± standard deviation. (n): number of cells used for these measurements.

| Species  (morphotype) | Morphotype | Major axis  (µm) | Minor axis  (µm) | Aspect  ratio | Circularity | n |
| --- | --- | --- | --- | --- | --- | --- |
|  |  |  |  |  |  |  |
| *T. angustilobata* | E | 7.2 ± 1.2 | 3.3 ± 0.6 | 2.2 ± 0.4 | 0.7 ± 0.1 | 114 |
| “ | S | 4.9 ± 0.7 | 4.2 ± 0.6 | 1.2 ± 0.1 | 1.0 ± 0.0 | 49 |
| *T. decolorans* | E | 7.5 ± 1.3 | 3.3 ± 0.6 | 2.4 ± 0.5 | 0.7 ± 0.1 | 136 |
| “ | S | 4.7 ± 0.6 | 4.0 ± 0.6 | 1.2 ± 0.1 | 0.9 ± 0.0 | 92 |
| *T. gelatinosa* | E | 8.2 ± 1.5 | 3.2 ± 0.6 | 2.6 ± 0.6 | 0.7 ± 0.1 | 122 |
| “ | S | 4.6 ± 0.7 | 3.7 ± 0.6 | 1.2 ± 0.1 | 0.9 ± 0.0 | 51 |
| *T. vagua* | E | 4.0 ± 1.3 | 1.9 ± 0.8 | 2.2 ± 0.3 | 0.7 ± 0.1 | 104 |
| “ | S | 2.7 ± 0.9 | 2.1 ± 0.9 | 1.3 ± 0.1 | 0.9 ± 0.0 | 59 |

**Tab. S2.** Presence of nine meiotic genes (i.e. dmc1, hop1, hop2, mer3, mnd1, msh4, msh5, rec8, and spo11; Schurko & Logsdon 2008, Fučíková *et al.* 2015) across 62 assembled Trebouxiophyceae genomes and one *Trebouxia gelatinosa* transcriptome. Numerical values represent the relative length of the obtained sequence compared to the reference sequence. Data referring to *Trebouxia* species are shown in bold.

| **TAXA** | **NCBI REFERENCE** | **ORDER** | **DMC1** | **HOP1** | **HOP2** | **MER3** | **MND1** | **MSH4** | **MSH5** | **REC8** | **SPO11** |
| --- | --- | --- | --- | --- | --- | --- | --- | --- | --- | --- | --- |
| *Apatococcus fuscideae* | GCA_040144375.1 | Chlorellales | 0.97 | 0.86 | 0.97 | 1.00 | 1.00 | 0.69 | 1.00 | 0.00 | 1.00 |
| *Apatococcus lobatus* | GCA_040144355.1 | Chlorellales | 1.00 | 0.86 | 0.00 | 0.90 | 1.00 | 0.29 | 1.00 | 0.00 | 0.90 |
| *Auxenochlorella protothecoides* | GCA_000733215.1 | Chlorellales | 0.97 | 0.85 | 0.95 | 0.00 | 0.98 | 0.72 | 0.00 | 0.00 | 1.00 |
| *Auxenochlorella pyrenoidosa* | GCA_001430745.1 | Chlorellales | 1.00 | 0.81 | 0.00 | 1.00 | 1.00 | 1.00 | 0.00 | 0.00 | 0.85 |
| *Chlorella desiccata* (nom. nud.) | GCA_019044685.2 | Chlorellales | 1.00 | 0.00 | 1.00 | 1.00 | 1.00 | 1.00 | 1.00 | 0.00 | 1.00 |
| *Chlorella ohadii* | GCA_025026875.1 | Chlorellales | 1.00 | 0.80 | 0.00 | 0.48 | 1.00 | 1.00 | 1.00 | 0.00 | 1.00 |
| *Chlorella sorokiniana* | GCA_025917655.1 | Chlorellales | 1.00 | 0.80 | 0.00 | 1.00 | 0.54 | 1.00 | 1.00 | 0.00 | 1.00 |
| *Chlorella* sp. | GCA_029286985.1 | Chlorellales | 1.00 | 0.80 | 0.96 | 0.48 | 1.00 | 1.00 | 0.00 | 0.00 | 0.81 |
| *Chlorella* sp. | GCA_003063905.1 | Chlorellales | 1.00 | 0.87 | 0.82 | 0.89 | 0.99 | 0.73 | 1.00 | 0.00 | 0.89 |
| *Chlorella* sp. | GCA_002896455.3 | Chlorellales | 1.00 | 0.86 | 0.00 | 1.00 | 1.00 | 1.00 | 1.00 | 0.00 | 1.00 |
| *Chlorella* sp. | GCA_013372505.1 | Chlorellales | 0.97 | 0.77 | 0.00 | 0.90 | 1.00 | 1.00 | 1.00 | 0.00 | 1.00 |
| *Chlorella* sp. | GCA_009928355.1 | Chlorellales | 1.00 | 0.80 | 0.00 | 1.00 | 0.85 | 0.71 | 1.00 | 0.00 | 0.98 |
| *Chlorella* sp. | GCA_006782975.1 | Chlorellales | 1.00 | 0.74 | 0.00 | 1.00 | 1.00 | 1.00 | 1.00 | 0.00 | 0.95 |
| *Chlorella* sp. | GCA_004335735.1 | Chlorellales | 1.00 | 0.86 | 0.45 | 0.77 | 1.00 | 0.66 | 1.00 | 0.56 | 0.99 |
| *Chlorella* sp. | GCA_025772305.1 | Chlorellales | 0.87 | 0.00 | 0.89 | 1.00 | 0.54 | 0.73 | 1.00 | 0.00 | 0.99 |
| *Chlorella variabilis* | GCA_000147415.1 | Chlorellales | 0.98 | 0.84 | 0.00 | 1.00 | 1.00 | 1.00 | 1.00 | 0.00 | 1.00 |
| *Chlorella vulgaris* | GCA_023343905.1 | Chlorellales | 1.00 | 0.77 | 0.00 | 0.90 | 1.00 | 1.00 | 1.00 | 0.00 | 1.00 |
| *Helicosporidium* sp. | GCA_000690575.1 | Chlorellales | 0.99 | 0.00 | 0.00 | 0.24 | 0.54 | 0.29 | 0.00 | 0.32 | 0.90 |
| *Marvania coccoides* | GCA_963854735.1 | Chlorellales | 0.97 | 0.00 | 0.92 | 0.91 | 1.00 | 1.00 | 1.00 | 0.00 | 1.00 |
| *Micractinium conductrix* | GCA_002245815.2 | Chlorellales | 1.00 | 0.84 | 0.00 | 1.00 | 0.54 | 1.00 | 1.00 | 0.00 | 1.00 |
| *Micractinium* sp. | GCA_029339195.1 | Chlorellales | 1.00 | 0.81 | 0.00 | 1.00 | 1.00 | 1.00 | 1.00 | 0.00 | 1.00 |
| *Nannochloris* sp. | GCA_004335565.1 | Chlorellales | 0.99 | 0.00 | 0.84 | 0.00 | 0.96 | 0.00 | 0.00 | 0.86 | 1.00 |
| *Nannochloris* sp. | GCA_004335555.1 | Chlorellales | 1.00 | 0.00 | 0.88 | 1.00 | 1.00 | 0.30 | 0.00 | 0.85 | 1.00 |
| *Parachlorella kessleri* | GCA_001598975.1 | Chlorellales | 1.00 | 0.86 | 0.95 | 1.00 | 1.00 | 0.75 | 1.00 | 0.00 | 1.00 |
| *Parachlorella* sp. | GCA_035048795.1 | Chlorellales | 1.00 | 0.86 | 0.95 | 1.00 | 1.00 | 0.75 | 1.00 | 0.00 | 1.00 |
| *Prototheca bovis* | GCA_003612995.1 | Chlorellales | 1.00 | 0.46 | 0.92 | 0.24 | 1.00 | 0.28 | 0.00 | 0.00 | 0.49 |
| *Prototheca ciferrii* | GCA_003613005.1 | Chlorellales | 1.00 | 0.48 | 0.27 | 0.09 | 1.00 | 0.34 | 0.34 | 0.32 | 0.81 |
| *Prototheca cutis* | GCA_002897115.2 | Chlorellales | 1.00 | 0.78 | 0.98 | 0.00 | 1.00 | 0.29 | 0.00 | 0.32 | 1.00 |
| *Prototheca stagnorum* | GCA_002794665.1 | Chlorellales | 1.00 | 0.85 | 0.00 | 0.00 | 1.00 | 0.61 | 0.00 | 0.00 | 1.00 |
| *Prototheca wickerhamii* | GCA_031763795.1 | Chlorellales | 1.00 | 0.77 | 0.99 | 0.00 | 1.00 | 0.29 | 0.00 | 0.32 | 0.97 |
| *Deuterostichococcus epilithicus* | GCA_033439295.1 | Prasiolales | 1.00 | 0.83 | 0.95 | 1.00 | 1.00 | 0.78 | 1.00 | 0.00 | 0.99 |
| *Diplosphaera chodatii* | GCA_032358165.1 | Prasiolales | 0.97 | 0.82 | 0.94 | 1.00 | 1.00 | 0.80 | 0.79 | 0.52 | 1.00 |
| *Prasiola linearis* | GCA_032356305.1 | Prasiolales | 0.95 | 1.00 | 0.95 | 0.87 | 0.37 | 0.66 | 0.23 | 0.33 | 0.61 |
| *Tetratostichococcus* sp. | GCA_036584455.1 | Prasiolales | 1.00 | 0.00 | 0.95 | 1.00 | 1.00 | 0.80 | 1.00 | 0.00 | 1.00 |
| *Myrmecia bisecta* | GCA_040144395.1 | Trebouxiales | 1.00 | 0.85 | 0.94 | 0.91 | 1.00 | 0.76 | 1.00 | 1.00 | 0.99 |
| *Asterochloris erici* | GCA_019693375.1 | Trebouxiales | 1.00 | 0.00 | 0.96 | 0.91 | 0.98 | 0.29 | 0.31 | 0.53 | 1.00 |
| *Asterochloris* sp. | GCA_963969365.1 | Trebouxiales | 1.00 | 0.00 | 0.93 | 0.91 | 1.00 | 0.29 | 1.00 | 0.53 | 1.00 |
| *Symbiochloris irregularis* | GCA_040144405.1 | Trebouxiales | 1.00 | 0.83 | 0.90 | 0.84 | 1.00 | 0.76 | 1.00 | 0.00 | 1.00 |
| ***Trebouxia decolorans*** | **unpublished data** | Trebouxiales | **1.00** | **0.85** | **0.87** | **0.91** | **0.99** | **0.49** | **0.84** | **0.00** | **1.00** |
| ***Trebouxia gelatinosa***  **(genome)** | **GCA_000818905.1** | Trebouxiales | **0.93** | **0.85** | **0.00** | **0.00** | **1.00** | **0.70** | **0.00** | **0.00** | **0.91** |
| ***Trebouxia gelatinosa* (transcriptome)** | **SRR988248** | Trebouxiales | **0.97** | **0.86** | **0.94** | **0.13** | **1.00** | **0.70** | **1.00** | **0.00** | **0.94** |
| ***Trebouxia* sp.** | **unpublished data** | Trebouxiales | **1.00** | **0.85** | **0.87** | **0.48** | **1.00** | **0.49** | **0.84** | **0.00** | **1.00** |
| ***Trebouxia* sp.** | **GCA_008636185.1** | Trebouxiales | **1.00** | **0.84** | **0.90** | **0.48** | **1.00** | **0.81** | **1.00** | **0.00** | **1.00** |
| ***Trebouxia* sp.** | **GCA_040206735.1** | Trebouxiales | **1.00** | **0.84** | **0.90** | **0.91** | **1.00** | **0.76** | **1.00** | **0.00** | **0.99** |
| ***Trebouxia* sp.** | **GCA_040206755.1** | Trebouxiales | **1.00** | **0.84** | **0.90** | **0.48** | **1.00** | **0.74** | **1.00** | **0.00** | **1.00** |
| ***Trebouxia* sp.** | **GCA_040206745.1** | Trebouxiales | **1.00** | **0.84** | **0.00** | **0.48** | **1.00** | **0.78** | **1.00** | **0.00** | **0.99** |
| ***Trebouxia* sp.** | **GCA_002118135.1** | Trebouxiales | **1.00** | **0.85** | **0.99** | **0.91** | **1.00** | **0.78** | **1.00** | **0.00** | **1.00** |
| *Botryococcus braunii* | GCA_002005505.1 | Trebouxiophyceae incertae sedis | 1.00 | 0.86 | 0.94 | 1.00 | 1.00 | 0.72 | 1.00 | 1.00 | 0.99 |
| *Choricystis* sp. | GCA_958009055.1 | Trebouxiophyceae incertae sedis | 1.00 | 0.80 | 0.00 | 1.00 | 1.00 | 0.29 | 0.00 | 0.32 | 1.00 |
| *Coccomyxa* sp. | GCA_000812005.1 | Trebouxiophyceae incertae sedis | 1.00 | 0.85 | 0.94 | 0.89 | 1.00 | 0.77 | 1.00 | 0.00 | 1.00 |
| *Coccomyxa* sp. | GCA_020887355.1 | Trebouxiophyceae incertae sedis | 1.00 | 0.77 | 0.94 | 0.90 | 1.00 | 0.76 | 1.00 | 0.00 | 1.00 |
| *Coccomyxa subellipsoidea* | GCA_000258705.1 | Trebouxiophyceae incertae sedis | 1.00 | 0.77 | 0.94 | 0.90 | 1.00 | 0.81 | 1.00 | 1.00 | 1.00 |
| *Coccomyxa viridis* | GCA_964019345.2 | Trebouxiophyceae incertae sedis | 1.00 | 0.84 | 0.94 | 0.89 | 1.00 | 0.78 | 1.00 | 0.00 | 1.00 |
| *Elliptochloris bilobata* | GCA_040144335.1 | Trebouxiophyceae incertae sedis | 1.00 | 0.86 | 0.98 | 0.90 | 1.00 | 0.72 | 1.00 | 0.00 | 1.00 |
| *Medakamo hakoo* | GCA_026636295.1 | Trebouxiophyceae incertae sedis | 1.00 | 0.86 | 0.00 | 0.00 | 1.00 | 0.29 | 0.00 | 0.00 | 1.00 |
| *Picochlorum costavermella* | GCA_011316045.1 | Trebouxiophyceae incertae sedis | 1.00 | 0.00 | 0.84 | 0.00 | 1.00 | 0.41 | 0.00 | 0.90 | 1.00 |
| *Picochlorum* sp. | GCA_009650465.1 | Trebouxiophyceae incertae sedis | 1.00 | 0.00 | 0.88 | 0.84 | 1.00 | 0.29 | 0.00 | 0.00 | 1.00 |
| *Picochlorum* sp. | GCA_025209345.1 | Trebouxiophyceae incertae sedis | 1.00 | 0.00 | 0.88 | 0.00 | 1.00 | 0.97 | 0.00 | 0.00 | 1.00 |
| *Picochlorum* sp. | GCA_010909725.1 | Trebouxiophyceae incertae sedis | 1.00 | 0.00 | 0.94 | 0.00 | 1.00 | 0.00 | 0.00 | 0.00 | 1.00 |
| *Picochlorum* sp. | GCA_002818215.1 | Trebouxiophyceae incertae sedis | 1.00 | 0.00 | 0.00 | 1.00 | 1.00 | 0.00 | 0.00 | 0.00 | 1.00 |
| Trebouxiophyceae sp. | GCA_003568905.1 | Trebouxiophyceae incertae sedis | 1.00 | 0.86 | 0.95 | 1.00 | 1.00 | 0.78 | 1.00 | 0.56 | 1.00 |
| *Chloroidium* sp. | GCA_004335625.1 | Watanabeales | 1.00 | 0.87 | 1.00 | 1.00 | 1.00 | 0.80 | 1.00 | 0.00 | 0.99 |
| *Chloroidium* sp. | GCA_004335615.1 | Watanabeales | 1.00 | 0.87 | 0.00 | 1.00 | 1.00 | 0.80 | 1.00 | 0.00 | 1.00 |
